# Impact of the Post-Harvest Period on the Chemical and Sensorial Properties of *planifolia* and *pompona* Vanillas

**DOI:** 10.3390/molecules29040839

**Published:** 2024-02-14

**Authors:** Anaïck Ravier, Pauline Chalut, Saida Belarbi, Cyrille Santerre, Nadine Vallet, Zeineb Nhouchi

**Affiliations:** Institut Supérieur International du Parfum, de la Cosmétique et de l’Aromatique Alimentaire (ISIPCA), 34-36 Rue du Parc de Clagny, F-78000 Versailles, France

**Keywords:** *Vanilla planifolia*, *Vanilla pompona*, gas chromatography, sensory analysis

## Abstract

Vanilla production in Guadeloupe is expanding. The main species grown is *Vanilla planifolia*, but other species such as *Vanilla pompona* are also present and required by industries. To upgrade the value of vanilla production on this Caribbean Island, this study was performed to evaluate the aromatic specifies of these vanilla species according to the length of the post-harvest period (2 months and 9 months). For this purpose, *Vanilla planifolia* and *Vanilla pompona* were compared through scald and scarification transformation processes, as well as two different refining times (T1 and T2). For chemical characterization, 0.1 g of vanilla bean seeds was used for SMPE/GC-MS measurements, while 0.05 g of vanilla samples was subjected to infusion in milk (0.15%) for sensory evaluation. The latter involved generation of terms of aroma through olfaction and gustation sessions. The chemical results showed a significant difference between the two species, where vanillin was mostly present in *Vanilla planifolia*, unlike *Vanilla pompona*, where it was mainly rich in 4-methoxybenzyl alcohol. Interestingly, the second refining time was characterized by the appearance of two major components, 1,3-octadien and acetic acid. For sensory analysis, all the vanillas exhibited a high diversity of aromas including “***sweet***”, ”***gourmand***”, “***spicy***” flavors and so on. The application of factorial correspondence analysis (FAC) as well as the agglomerative hierarchical clustering (AHC) showed differences between the vanilla samples according to both the species and refining time. The combination of these analyses makes it possible to establish a chemical and organoleptic profile of vanillas. Varietal and processing factors both have a major impact on the aroma profile of vanillas.

## 1. Introduction

Vanilla, historically called black flower, was initially discovered in Mexico (1427–1440) and used essentially to flavor a noble cocoa drink. It was introduced in Europe in the Paris botanical garden in 1793. Then, it thrived on Reunion Island and in Madagascar (1822–1850), followed by Indonesia, the Philippines, and Tahiti (1846–1848), due to their tropical climates [1]. Based on these different origins and climatic conditions, vanilla has today a spectacular botanical variability with 18,500 species across 788 genera [2]. Among this huge number of species, three of them are cultivated: *Vanilla planifolia*, *Vanilla pompona*, and *Vanilla tahitensis*, where *Vanilla planifolia* accounts for about 95% of global commercial production [3].

Despite this important number of species and significant production, vanilla still ranks as the second most costly spice worldwide following saffron [4]. Its high price may be explained by the pollination of the flowers that occurs manually as well as the laborious and long beneficiation of the fruits [5]. Moreover, its preeminent position in the global market is also explained by its high quality of flavoring. For instance, in the food industry, vanilla is omnipresent in the formulation of ice cream, yogurt, baked products, and so on, due to its delicious taste and pleasant flavor [3].

Considering cosmetics, vanilla extracts are mainly added to the products as a flavoring agent and have recently shown great antioxidant properties for the skin. Regarding its fine fragrance, vanilla is a key player in fragrance creation, enhancing olfactory experiences by seamlessly merging with caramel, amber, and elusive floral notes like lily. In the intricate tapestry of perfumery, vanilla emerges as an indispensable and versatile note, contributing to diverse accords that compose captivating olfactory masterpieces [4]. Most of these goods are flavored with vanillin (4-hydroxyl-3-methoxybenzaldehyde), known as the main molecule of vanilla extracts, obtained by various chemical and biotechnological processes [6].

In this context, the literature review showed that various analytical techniques have been used for many years to investigate the chemical profile of vanilla. The most classical methodology is gas chromatography coupled with various detection sources. For example, Chen et al. [7] identified 69 volatile compounds using mass spectrometry (GC-MS) to detect phenols, ketones, alcohols, aldehydes, esters, and acids. Yeh et al. [8] used GC-MS and GC coupled with a flame ionization detector (GC-FID) to characterize the volatile compounds of vanilla produced in Taiwan, highlighting the differences linked to the growing conditions. GC hyphenated to Olfactometry (GC-O) is also a very interesting tool for characterizing odorous compounds provided by vanilla beans [7].

In addition to the origin, sample preparation is also a key factor impacting the chemical composition of vanilla extracts. For instance, Chen, Lin, Lo, and Hsu [7] used ethanol to prepare extracts for subsequent analysis, while Yeh et al. compared the analysis of alcoholic extracts with the Head Space technique coupled with Solid Phase Micro-Extraction (HS-SPME). This second technology was presented as the most efficient in their study. In another approach, Hartman et al. [9] described how the use of Thermal Desorption (TD-GC) can be used to identify 60 flavor compounds, where 18 of them were phenolic compounds. Moreover, Da Costa and Pantini [10] identified 276 compounds using a combination of different sample preparation techniques. The use of Dynamic HeadSpace (DHS) (GERSTEL, Mülheim an der Ruhr, Germany) revealed the presence of 79 of these compounds [10]. Direct analyses such as Selected Ion Flow Tube Mass Spectrometry (SIFT-MS) are described in the work of Langford et al. [11], and they were considered as selective analysis with good sensitivity. Pitman and LaCourse [12] used Molecular Ionization Desorption Analysis Source (MIDAS) as a rapid analysis method, particularly for fraud identification. Near-infrared spectroscopy (NIR) is also an interesting technology for the analysis of volatile compounds in vanilla as reported by Budriastra et al. [11]. While instrumentation can effectively measure and identify volatile compounds, comprehending the nuanced sensory attributes of vanilla as experienced by humans is a much more intricate task. Indeed, identifying the key components responsible for the organoleptic characteristics of vanilla is an important issue (i) to better describe this specific flavor and (ii) to better valorize it among consumers and their requirements [13].

To discern the sensory profiles of vanilla products, it is necessary to use sensory analysis techniques. Indeed, the commercial value of vanilla is related to its sensory proprieties, particularly the taste and the flavor provided by the cured pods. The most perceived attributes of vanilla beans include descriptors such as “***sweet***”, “***vanillin***”, “***floral***”, “***spicy***”, “***woody***”, and “***tobacco-like***”. These latter are not only associated with vanillin. Indeed, vanillin itself presents only 12% of the vanilla pod composition [14]. Interestingly, an average of 205 minor compounds also contribute to the sensory profile of this spice and differ between vanilla samples according to their concentrations, which may be related to the production process and the growth conditions of the plant such as p-hydroxybenzaldehyde (***sweet***), vanillic acid (***chocolate***, ***creamy***, ***grape***, ***nutty***, and ***wine-like***), p-hydroxybenzoic acid (**phenolic**), and vanillyl alcohol (*mild*, *sweet*, *balsamic*, and *vanilla-like odor*) [15,16].

That is why the sensory profile of vanilla cannot be defined as one-dimensional. It is subtle and complex. It may have many dimensions [17]. Indeed, volatiles are more concerned with olfactory receptors, whereas non-volatile components are more concerned with taste receptors; consequently, both have different effects on flavor and aroma. Clearly, it is interesting to investigate both the taste and odor impact of vanilla in sensorial perceptions.

As shown above, the main studied factors of the aromatic profile of vanilla are the region, the transformation processes, the sample preparations, and the chemical instrument. However, the post-harvest period is not well studied. Moreover, the literature data showed that the taste and olfactory perceptions of vanilla are mainly studied separately. Few research papers investigate both features.

With this lack of information, it is crucial to comprehend the influence of the sensory profiles derived from various vanilla bean origins on the ultimate olfactive and taste sensory experience based on the post-harvest period. For this purpose, this present study provides significant comparisons.

## 2. Results

### 2.1. Chemical Aspect

#### 2.1.1. Chemical Analysis

Analysis of the different vanillas by HS-SPME-GC-MS revealed the presence of at least ten different chemical families. This chemical diversity includes alcohols, aldehydes, ketones, phenols, esters, alkenes, furanic compounds, etc. The most common families are alcohols, aldehydes, and esters (Table 1). Chemical analyses revealed differences in chemical composition between the vanilla species, scarified and scalded vanilla, and different refining times. For instance, 1,3-octadien, appeared only in the second refining time in all three samples. This is also the case for acetic acid. Similarly, acetoin was observed at the second refining time only in *Vanilla planifolia*. Regarding carvone, it was identified at the second refining stage in *Vanilla pompona* and scarified *Vanilla planifolia*. *Vanilla pompona* contained benzyl alcohol and ethyl phenyl alcohol at the first stage of refining, in contrast to *Vanilla planifolia*. This was also the case for benzaldehyde and benzyl acetate. In contrast, compounds such as hexanal were present only in the first stage of refining and disappeared in the second. The same is true of limonene, which was present in the first stage of refining in scalded *Vanilla planifolia* and disappeared in the second. As for vanilla’s characteristic molecule, vanillin, it was present in higher quantities in scalded *Vanilla planifolia* at the first stage of refining than in *Vanilla pompona*. The same applied to the second stage of refining, but the values were closer between scalded *Vanilla planifolia* and *Vanilla pompona*.

#### 2.1.2. Statistical Analysis

**Figure 1:**
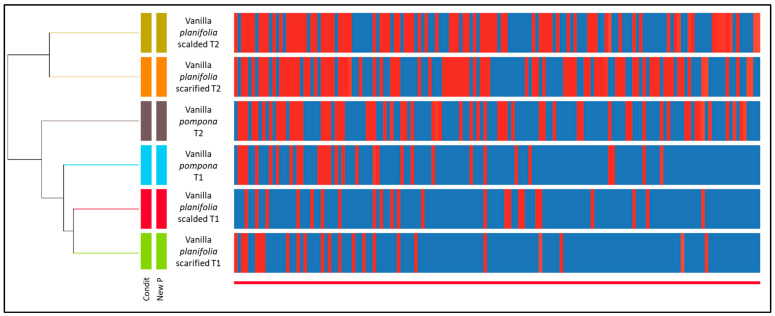
Chemical classification of all vanillas, considering both refining times obtained by the application of agglomerative hierarchical clustering (AHC).

Statistical analysis of the GC-MS data using Hierarchical Ascending Classification (HAC) revealed different clusters (Figure 1). At T1, *Vanilla planifolia* vanillas were similar in terms of the chemical composition, whatever the method used, i.e., scarified or scalded. At the same time, there was a real differentiation between *Vanilla pompona* and the two *Vanilla planifolia* types.

At T2, *Vanilla pompona* remained close to *Vanilla pompana* at T1, indicating little chemical evolution in the chromatographic profile. At T2, the *Vanilla planifolia* group really stood out compared with *Vanilla planifolia* T1 and the two *Vanilla pompana* times. This shows that the maceration time significantly changed the chemical profile of this type of vanilla. On the other hand, the two scarified or scalded methods did not show a real differentiation in chemical profiles as in T1, and these two *Vanilla planifolia* remained similar.

### 2.2. Sensory Aspect

#### 2.2.1. Olfactory Results

##### Flavor Perception Based on Descriptors Frequencies

Table 2 presents the frequency of the terms generated by the panel. Regardless of the type of vanilla species, a total of 634 terms were provided by assessors for the first refining time according to an ascending order, where there were 182 terms for scalded *Vanilla planifolia*, 216 terms for scarified *Vanilla pompona*, and 236 terms for scarified *Vanilla planifolia*. Fewer terms were generated for the second refining time describing the olfactory profiles of all vanillas (491 terms). Interestingly, the same order was obtained for the second refining time. Indeed, 150 terms were provided by assessors to qualify scalded *Vanilla planifolia*, while there were 165 terms for scarified *Vanilla pompona*, followed by 176 terms for scarified *Vanilla planifolia*.

Regardless of the refining time, it is noticeable that the numbers of terms generated for both scarified *Vanilla pompona* and for scarified *Vanilla planifolia* were close and significantly higher than the number of attributes provided by assessors for the scalded *Vanilla planifolia*. This result may emphasize the impact of the transformation process on the olfactory profile of vanilla rather than the type of species. Indeed, for the studied samples, the scarification process includes a drying step in the rising and setting sun for three days. However, the scalding transformation involved only twenty-four hours of drying in the sun. It is well known that heat treatment (exposure to the sun in the present study) is important for generating odors.

To evaluate the performance of the panel, the number of different terms was identified for both refining times. The first time, out of 634 terms generated by participants, approximately 27% of them were different (171 terms). Regarding scalded *Vanilla planifolia*, approximately 25% of the generated terms were different (42 out of 171), with 32% for scarified *Vanilla planifolia* and 43% for scarified *Vanilla pompona*. Similar findings were obtained for the second refining time, where approximately 38% of variability was found (187 different words out of 491), following this order: 28% for scalded *Vanilla lanifolia*, 33% for scarified *Vanilla planifolia*, and 38% for scarified *Vanilla pompona*.

Based on this result, the impact of the species was evident, since the closest percentages were found for both scalded *Vanilla planifolia* and scarified *Vanilla planifolia*. Moreover, an agreement and consensus between participants could be suggested since the percentage difference between participants was found to be less than 50%.

##### Generation of Terms 

To elaborate the olfactive profile and compare the impact of the transformation process, common descriptors provided by assessors after smelling vanilla beans were identified and are illustrated in Figure 1a. Regardless of the vanilla species and the refining time, all the vanilla samples were perceived as “***gourmand***”, “***vanilla***”, “***milky***”, “***woody***”, “***roasted***”, “***spicy***”, “***nutty***”, “***green***”, “***floral***”, “***animal***”, “***moist woody***”, “***alcoholic***”, and “***fruity***”.

The frequency of each descriptor depended strongly on the vanilla type as well as the refining time. For example, the “***gourmand***” attribute was cited 61 times for the scalded *Vanilla planifolia*, followed by 58 times for the scarified *Vanilla planifolia* and 54 times for scarified *Vanilla pompona* at the first refining time. These frequencies decreased significantly for the second refining time, and the order was changed. Indeed, the scarified *Vanilla pompona* exhibited the highest frequency of gourmand at 51 times, followed by scarified *Vanilla planifolia* (45 times) and scalded *Vanilla planifolia* (44 times).

To establish the aromatic profiles of the vanilla samples, only descriptors with frequencies higher than 10 times were considered. Thus, the scalded *Vanilla planifolia* was mostly characterized by “***gourmand***”, “***vanilla***”, “***milky***”, “***woody***”, “***nutty***, “***roasted***”, and “***spicy***” attributes. However, the scarified *Vanilla planifolia* was perceived as “***animal***”, “***vanilla***”, “***spicy***”, “***gourmand***”, “***nutty***”, “***milky***”, “***fruity***”, “***moist woody***”, and “***roasted***”. Regarding the scarified *Vanilla pompona*, the main descriptors were, “***gourmand***”, “***vanilla***”, “***milky***”, “***animal***”, “***spicy***”,” ***floral***”, “***nutty***”, and “***woody***”.

For the first refining time, the “***moist woody***” descriptor was present for all vanilla. This result could be associated with the moisture content of the samples and the short period of transformation (two months) after harvest. Interestingly, the “***moist woody***” descriptor disappeared at the second time of transformation for both scarified *Vanilla planifolia* and scarified *Vanilla pompona* since the period of transformation after harvest was longer (9 months), allowing the reduction in moisture. For the scalded *Vanilla planifolia*, the moist woody descriptor was evocated by assessors for both refining times since the scalding process included a moisturizing step.

Furthermore, the second refining time was found to enhance the “***vanilla***” (close frequencies ranging from 37 to 44), “***gourmand***” (close frequencies ranging from 43 to 51), “***animal***” (close frequencies ranging from 13 to 15), and “***spicy***” (close frequencies ranging from 13 to 21) descriptors regardless of the species and the transformation process.

##### Statistical Classification of the Vanilla Samples

Multivariate statistical analysis, FCA, was performed on the descriptors and the vanilla samples to highlight the similarities between samples and their proximities with the aroma. The terms that were used less than 15% in frequency were not retained for FCA analysis. The two factors of the FCA were considered for each refining time.

**Figure 2:**
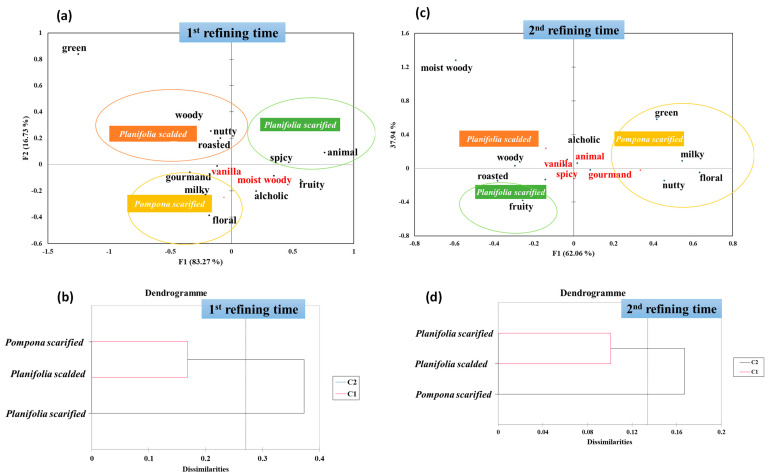
(**a**,**c**) Factorial correspondence analysis (FCA) similarity map and (**b**,**d**) classification of vanilla samples obtained by the application of agglomerative hierarchical clustering (AHC) applied for the olfactive results for both the first and second refining times.

Regarding the first refining time (Figure 2a), the first two factors of the FCA explained 100% of the variance (F1 = 83.27% and F2 = 16.73%). These factors added greatly to the total variance; so, they were considered for further statistical analysis. F1 separated scalded *Vanilla planifolia* and scarified *Vanilla pompona* in the negative direction, from scarified *Vanilla planifolia*. The weighting of this axis was less significant than the F1 and did not show any tendency regarding the transformation process in classifying the vanilla samples. However, along the F1 axis, there was a clear separation of vanilla according to the species, where the scarified *Vanilla planifolia* and scalded *Vanilla planioflia* moved in the positive direction and the scarified *Vanila pompona* in the negative direction.

Furthermore, the application of the FCA analysis showed that the descriptors could be separated into three distinct groups according to the vanilla samples. Indeed, the map showed that the scalded *Vanilla planifolia* was associated with the descriptors “***woody***”, “***nutty***”, and “***roasted***”. However, the scarified *Vanilla pompona* was assigned to “***gourmand***”, “***milky***”, and “***floral***” aromas. The last group containing scarified *Vanilla planifolia* was characterized by “***green***” and “***spicy***”. Interestingly, the “***vanilla***” and “***moist woody***” terms were centered in the map, showing that these attributes were common between vanilla samples.

In addition to the FCA analysis, the AHC was generated to analyze the preprocessed data (Figure 2). This analysis allows us to highlight the proximities and differences between the vanilla samples. First, the number of clusters was set to be equal to three, displayed graphically using a tree diagram, known as a dendrogram, which showed all the steps in the hierarchical procedure.

Considering the first refining time, the resulting dendrogram clearly divided the vanilla samples into two groups, regarding the proximity between them.

**Figure 3:**
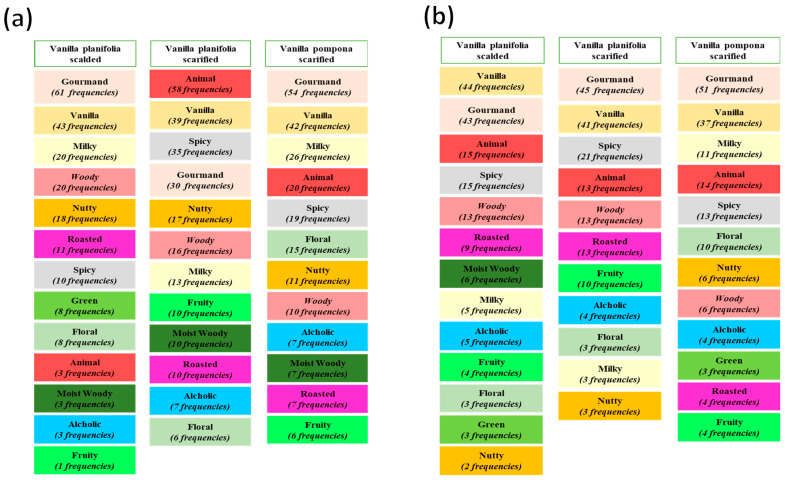
Frequencies summary for the most characteristic flavors of vanilla samples for (**a**) the first and (**b**) the second refining times.

The first group was composed of both scarified Vanilla pompona and scalded *Vanilla planifolia*, which enhanced the distribution of these species, considering F2 in the FCA map. This result is in line with the olfactive profiles of these two samples. Indeed, both exhibited high frequencies for the “***gourmand***”, “***vanilla***”, and “***milky***” descriptors, as shown in Figure 3. The second group was formed only by the scarified *Vanilla planifolia*. The distance between this sample and the first group could be explained by the “***animal***” and “***spicy***” attributes that were highly cited by assessors.

For the second refining time, a similar discrimination was obtained when considering the vectors. Indeed, the first vector F1 (62.06% of the total variance) and the second one F2 (37.94% of the total variance) discriminated mostly between the vanilla samples according to the species, as observed for the first refining time (Figure 2c). Indeed, considering the first axis, F1, the scarified *Vanilla pompona* was on the positive side, and scarified *Vanilla planifolia* and scalded *Vanilla planifolia* exhibited negative scores and were placed on the negative side. Regarding the F2, no tendency according to the species and the type of transformation was observed.

Considering the distance between the vanillas samples projected in the FCA map, the most qualifying attributes of each sample were highlighted. Indeed, the scalded *Vanilla planifolia* was particularly “woody” and “***moist woody***” in agreement with the results obtained in the terms generation session. The scarified *Vanilla planifolia* was found to be “***fruity***” and “***roasted***”, while the Vanilla planifolia scalded was more “***nutty***”, “***floral***”, “***milky***”, and “***green***”. Interestingly, the descriptors “***vanilla***”, “***spicy***”, “***gourmand***”, and “***animal***” were projected in the center of the map, in agreement with the previous results.

Similar to the first refining time, the AHC and the obtained dendrogram were analyzed (Figure 2b,d). Indeed, the scarified *Vanilla planifolia* and scalded *Vanilla planifolia* belonged to the same group, while the scarified *Vanilla pompona* formed a separate group. This result enhances the impact of the refining time in the maturation of the aromatic profile of vanilla.

#### 2.2.2. Taste Results

##### Flavor Perception Based on Descriptor Frequencies

Table 3 presents the frequency of taste descriptors provided by assessors. Generally, the number of terms generated for taste evaluation (772 terms for the first refining time and 595 terms for the second refining time) was significantly higher than those provided for the olfaction tests (634 terms for the first refining time and 491 terms for the second refining time). This result could be explained by the physiological mechanisms of taste and olfaction perceptions. Indeed, the taste mechanism involves both mouth and nose.

Astonishingly, a similar trend was observed for the generation of terms of taste evaluation in comparison with the olfaction tests.

Indeed, considering the first refining time, a total of 772 terms were enumerated, with 236 terms for scalded *Vanilla planifolia*, 255 terms for scarified *Vanilla pompona*, and 281 terms for scarified *Vanilla planifolia*. Fewer terms were generated for the second refining time describing the taste sensory profiles of all vanillas (595 terms). Interestingly, the same order was obtained for the second refining time. Indeed, 196 terms were provided by assessors to qualify scalded *Vanilla planifolia*, while there were 198 terms for scarified *Vanilla pompona*, followed by 201 terms for scarified *Vanilla planifolia*.

Moreover, a smaller number of descriptors was observed for scalded *Vanilla planifolia*, similar to the olfactory test, confirming the importance of a long period of drying for the maturation and volatilization of flavors. Identical to the olfaction sessions, the panel was found to be homogeneous with a good consensus, since the difference of terms at the first refining time ranged from 31% to 37% and from 32% to 35% for the second refining time.

##### Generation of Terms 

**Figure 4:**
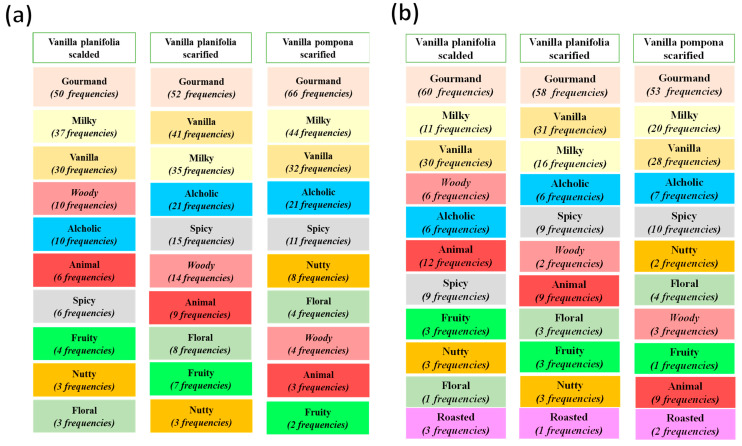
Frequencies summary for the most characteristic taste descriptors of vanilla samples for (**a**) the first and (**b**) the second refining time.

Similarly, assessors described the taste perception of vanilla beans prepared in hot milk. The most relevant descriptors were “***gourmand***”, “***vanilla***”, “***milky***”, “***woody***”, “***spicy***”, “***nutty***”, “***floral***”, “***animal***”, “***alcoholic***”, and “***fruity***”. Three descriptors were not present, “***green***”, “***moist woody***”, and “***roasted***” as showed in Figure 4. This finding could be explained by the threshold perception of these flavors. They are more perceived by olfaction than taste.

Surprisingly, the “***alcoholic***” descriptor was more present in the taste qualification of perceptions. This finding is related to the sensitivity of the physiological mechanism of taste in identifying this component. It could be also associated with the threshold of the components responsible for this perception. Indeed, it could be suggested that this attribute is mostly detected by taste rather than olfaction.

Regardless of the refining time, the most cited descriptors (>15 times) for scalded *Vanilla planifolia*, scarified *Vanilla planifolia*, and scarified *Vanilla pompona* were “***gourmand***”, “***vanilla***”, “***milky***”, “***alcoholic***”, and “***spicy***”. The frequencies of these descriptors were more homogenous and presented the same order for the second refining time. This result could be explained by the impact of the long period before harvest (9 months) that seems to help the maturation of the aromatic profile of vanilla samples.

Furthermore, the second refining time was found to generate a supplementary descriptor, “***roasted***”, for all the vanilla samples.

##### Statistical Classification of the Vanilla Samples

**Figure 5:**
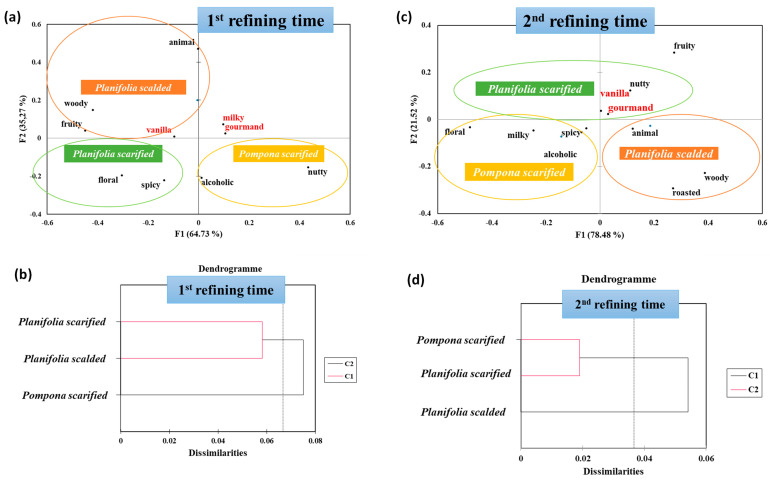
(**a**,**c**) Factorial correspondence analysis (FCA) similarity map and (**b**,**d**) classification of vanilla samples obtained by the application of agglomerative hierarchical clustering (AHC) applied on taste results for the first and second refining times.

Regarding the first refining time, the first two factors of the FCA explained 100% of the variance (F1 = 64.73% and F2 = 35.27%) separating well the vanilla samples according to both the species and the transformation process (Figure 5a). Indeed, F1 discriminated scalded *Vanilla planifolia* and scarified *Vanilla planifolia* from scarified *Vanilla pompona*. The weighting of this axis is significant since it separated samples according to the species.

This result was enhanced by the application of ACH, where scalded *Vanilla planifolia* and scarified *Vanilla planifolia* belonged in the same group.

Regarding F2, there was a clear separation of vanilla according to the transformation process, where the scarified *Vanilla planifolia* and scarified *Vanilla pompona* were on the negative side, and the scalded *Vanilla planioflia* was on the positive side.

Moreover, the FCA was applied to associate the descriptors with the vanilla samples. The projection on the FCA map showed that the scalded *Vanilla planifolia* was mostly “***woody***”, “***animal***”, and “***fruity***”, corroborating the previous results of olfaction, where this vanilla was qualified also as “***woody***”. However, the scarified *Vanilla pompona* was assigned to “***alcoholic***” and “***nutty***” aromas. It could be suggested that the moisturizing step favored the alcoholic perception after the scalding transformation.

Regarding scarified *Vanilla planifolia*, the most relevant descriptors were “***floral***” and “***spicy***”, greatly in concordance with the results of the olfaction session. Interestingly, the “***vanilla***”, “***milky***”, and “***gourmand***” terms were centered in the map, showing that these attributes were common between vanilla samples.

For the second refining time, the best discrimination was obtained when considering the first vector F1 (78.48% of the total variance) (Figure 5c). Considering this axis, the scarified *Vanilla pompona* and the scarified *Vanilla planifolia* exhibited negative scores, while scalded *Vanilla planifolia* was on the positive side according to the transformation process. This result was confirmed by the application of ACH showing the similarity between the scarified vanillas.

The comparison of the descriptors associated with the vanilla samples using the FCA map showed an interesting result. Indeed, identical to the first refining time, the scalded *Vanilla planifolia* was mostly “***woody***” and “***roasted***”. Additionally, the scarified *Vanilla pompona* was assigned to “***alcoholic***”, while the scarified *Vanilla planifolia* was particularly “***nutty***” and “***gourmand***”.

Similar to the first refining time, the AHC and the obtained dendrogram were analyzed (Figure 5b,d). Indeed, the scarified *Vanilla planifolia* and scalded *Vanilla planifolia* belonged to the same group, while the scarified *Vanilla pompona* formed a separate group. This result emphasizes the impact of the refining time in the maturation of the aromatic profile of vanilla.

## 3. Discussion

Today, consumers tend to make their purchase decisions of food, cosmetics, and fragrance based on sensory qualities, such as taste, mouth feel, skin feel, aroma, and so on. That is why industries have developed a strong interest in aromatic compounds to introduce pleasing perceptions and to satisfy consumers’ requirements [18]. Natural vanilla is within the scope of these issues. Indeed, this raw material has an important spectrum of aromatic compounds, which may diversify its applications. It constitutes one of the most preferred flavors and fragrance ingredients in ice cream, confectioneries, milk products, perfumes, pharmaceuticals, liqueur, and other cordial industries, thereby forming a multimillion-dollar market [19]. According to the literature, many factors can vary vanilla compositions such as the harvest period, regions, species, and so on.

In this context, this study has been performed to contribute to the enhancement of the scientific community with results including the chemical and sensorial characterization of vanilla beans for a better understanding of these raw materials according to different factors, notably, the species, the post-harvest period, and the transformation process. It could also provide some guidelines for industries wishing to flavor products with natural vanilla. On the one hand, the use of HS-SPME-GC-MS identified a rich diversity of common chemical families in all the vanilla samples, including alcohols, aldehydes, ketones, phenols, esters, alkenes, and furanic compounds. These molecules have been identified already in the literature. Indeed, since the last century, identification of the chemical components of vanilla has attracted considerable attention, and more than 200 compounds have been identified including tannins, polyphenols, free amino acids, and resins [16]. Regarding the aromatic composition, vanilla shows variations in its aromatic profile associated with each species and the curing process to which it is subjected, such as 1,3-octadien and acetic acid, as well as benzyl alcohol, ethyl phenyl alcohol, benzaldehyde, and benzyl acetate.

On the other hand, some chemical samples have been observed with different concentrations, depending on the vanilla species as well as the refining time. These molecules are para-anisyl alcohol, nonanal, para-anisaldehyde, methyl salicylate, and vanillin (Table 4). The latter is the major compound influencing the vanilla flavor, contributing 1–2% *w*/*w* of the cured pod; however, the other additional minor compounds from the pods also contribute and are responsible for the differences between the vanilla flavor and pure chemical vanillin. The aroma of cured vanilla beans includes descriptors providing many sensorial perceptions, such as “***sweet***”, “***vanillin***”, “***floral***”, “***prune***/***raisin***”, “***spicy***”, “***woody***”, “***nutty***”, “***floral***” and “***fruity***”, and “***tobacco***-***like***” [18], in accordance with the sensorial results obtained in this present study. Indeed, the sensorial characterization of vanilla beans with a panel of 50 assessors showed that these raw materials have been mainly perceived as “***gourmand***”, “***vanilla***”, “***milky***”, and “***spicy***” for both the taste and olfaction sessions. These findings may suggest a tendency of a correlation between the chemical composition and sensorial profiles [13]. This side should be greatly explored with a rich database of characterization to provide a suitable model, helping researchers to determine the aromatic profile of the vanilla base on the region, the species, the transformation, and the post-harvest period.

Considering all these similarities and dissimilarities, it could be hypothesized that the applied process (scarification or scalding) favors the physicochemical, biochemical, and microbiological environment, which significantly impact tissues of the vanilla bean and thus its chemical composition and its sensory specificities, corroborating the findings of Januszewska, Giret, Clement, Van Leuven, Goncalves, Vladislavleva, Pradal, Nåbo, Landuyt, D’Heer, Frommenwiler, and Haefliger [5], who recommended highly the use of sustainable environmentally friendly biotechnological processes to obtain products with added value.

Indeed, the authors suggested that the folding and shrinkage of the mesocarp cells of vanilla, according to the transformation processes, could create a synergistic effect among enzymes, microorganisms, water activity, and humidity loss, providing the development of the molecules responsible for the vanilla aromatic profile. Additionally, the authors stated that scarifying or scalding could involve the waste of polysaccharides, sugars, and aromatic compounds. That is why they highly recommended the use of sustainable environmentally friendly biotechnological processes to obtain products with added value of sensorial perceptions of *Vanilla planifolia* and *Vanilla pompona.* The present study showed similarities with the results reported in the literature in terms of the attributes found by both Om et al. [20] and Januszewska, Giret, Clement, Van Leuven, Goncalves, Vladislavleva, Pradal, Nåbo, Landuyt, D’Heer, Frommenwiler, and Haefliger [5] despite the use of hot water as solvent instead of milk. For instance, vanilla beans were described by their assessors as “***phenolic***”, ***woody***”, “***powdery vanilla***”, “***almondy***”, “***anisic***”, “***powdery***”, “***fruity***”, “***beany***”, “***woody***”, and “***oily mouthfeel***”. This result suggests that the aromatic profile of vanilla depends strongly on the matrix, and the role of the solvent is to enhance the intensity of the perception, in agreement with the literature [21]. Thus, all studies can be compared without missing some of the sensorial dimensions of vanilla.

To conclude, the present study aimed to elucidate the interaction between the sensorial properties of vanilla and its chemical composition. No correlation was established between these parameters, which may be considered as a limitation for this paper. However, some tendencies highlighting the link between the species and the refining time were observed and may help the scientific community to understand the vanilla manifestation in technological applications. Furthermore, the impact of endophytic microorganisms as well as the bean physiological maturity on the final composition of vanilla and its aromatic profile were not explored in this paper. That is why further experiments and other projects dealing with vanilla characterization are required and could help industries as well as researchers to improve the quality of vanilla and its incorporation into commercial products. Additionally, all this information may help producers to improve their curing processes and to choose the best maturity stage for the obtention of a high quality of aromatic profile [22].

## 4. Materials and Methods

### 4.1. Raw Materials

The studied vanilla beans come from the French island Guadeloupe. They were supplied by the APAGWA association (Association de Promotion de l’Agriculture et du Développement Rural en Guadeloupe). Two species, *Vanilla pompona* and *Vanilla planifolia*, were subjected to chemical and sensory characterization. The *Vanilla pompona* variety is grown in Bouillante and the *Vanilla planifolia* in Sainte-Rose. These two species have been chosen for this study because they are the most cultivated varieties in the French island of Guadeloupe [23,24].

These varieties were compared at two maturation times, T1 (two months after harvest) and T2 (nine months after harvest), following two processing methods: scalding or scarification. The choice of these processes was based on the simulation of the real conditions of vanilla production according to the internal processing steps of the APAGWA association. Both of these processes allow the development of phenolic compounds and fatty and organic acids that contribute to the development of its aromatic profile, which may be related to morphological, structural, chemical changes, and endophytic microorganisms, which together interact to provide the appreciated aromatic profile. However, this has only been studied during the bean’s physiological maturity and at the end of the curing process.

For this purpose, in this study, two different stages post-harvest were chosen to elucidate the relationship between the aromatic profile of vanilla and maturity.

For scalding, the vanillas were scalded in water for three minutes and placed in wooden boxes lined with a woolen blanket for twenty-four hours. The beans were then dried in the sun for a whole day. The dried vanillas were stored in the shade to complete the maturing process.

### 4.2. Chemical Aspects

#### 4.2.1. HS-SPME/GC-MS Analysis

Volatile organic compounds (VOCs) were determined by the HS-SPME/GC–MS method. Approximately 0.1 g of vanilla bean seeds was added to a sealed headspace vial of 10 mL. The HS-SPME extractions were carried out using one-centimeter-long fibers coated with divinylbenzene/carboxen/polydimethylsiloxane (DVB/CAR/PDMS, 50/30 µm) purchased from Supelco (Bellefonte, PA, USA). All the fibers were conditioned prior to analysis, according to the manufacturer’s instructions. The SPME procedure was performed automatically using the Agilent CTC PAL autosampler (Agilent, Santa Clara, CA, USA) equipped with the GC-MS instrument. It consisted of the following sequential operations: sample conditioning under stirring for 150 s at 50 °C, sample extraction for 30 min, and injection by thermal desorption of the fiber.

A 7890A GC coupled with a 5977A MSD single quadrupole manufactured by Agilent (Santa Clara, CA, USA) was used to analyze the VOCs. A Supelco SLB^®^-5ms GC column 30 m in length, 0.25 mm in internal diameter, and with a 0.25 μm film thickness was used to separate the VOCs (Sigma-Aldrich, St. Louis, MO, USA). Helium was used as the carrier gas at a flow rate of 1 mL/min. The column temperature was programmed from 40 °C to 220 °C at 6 °C/min. The final temperatures were held for 15 min. The detector temperature was set to 270 °C. The total GC cycle time was 45 min. The transfer line and quadrupole temperatures were 250 °C and 230 °C, respectively. Electron ionization (EI) mass spectra were recorded at 70 eV in positive mode, in the range *m*/*z* 30–400, using full SCAN (Total Ion Chromatogram) mode. The data were collected using the GCMS Data Analysis software B.07.02.1938 and screened and matched with NIST and other dedicated standard spectrum libraries to identify each component of the VOCs. The mass spectrometer peaks that were considered for analyses were those that matched the library at least more than 80 percent. The peaks were identified by comparing the experimental data with the mass spectrometer library (HPCH 2205, ISIPCA and NIST14). This identification was completed by the linear retention index, calculated using *n*-alkanes (C7–C40) as a reference and retention indexes in an internal database.

#### 4.2.2. GC-MS Statistical Analysis

Data processing was performed using Agilent Mass Hunter Qualitative Analysis (version B.06.00). Peak integration and deconvolution (parameters were the same as previously reported, except the SNR threshold of 3.026) were performed on the Mass Hunter. All the GC-MS spectra were exported as CEF format and uploaded to Mass Profiler Pro (MPP) for peak alignment, normalization, significance testing, fold change, and multivariate analysis for both the identified and unidentified compounds.

All the available data (full scan mode from *m*/*z* 50 to 400 and retention time window 0 to 45 min) were scanned, and the minimum absolute abundance of 50,000 counts was used to filter the data. The alignment parameters were set as the retention time tolerance 0.3, match factor 0.3, and delta *m*/*z* 0.3. A total of 276 entities were found in the entire samples after alignment. The entities were filtered by frequency, *p* ≤ 0.001, fold change >3, and coefficient of variance (CV) < 25%. Numerous entities were found to be significantly different in the samples. Hierarchical clustering was performed by applying Pearson’s uncentered-absolute distance metric, complete linkage.

### 4.3. Sensory Analysis

All the sensory tests were performed at the sensory laboratory of ISIPCA. Testing and data collection took place in standard sensory booths, under white lighting, with controlled temperature (20 ± 2 °C) and airflow conditions.

#### 4.3.1. Free Term Generation Methodology

Fifty panelists (83% women, 17% men) aged from 18 to 61 years participated in this study. The assessors were qualified on aroma evaluation since they were students and employees of ISIPCA. They were subjected to five training sessions. The gender parity was not considered as a decisive criterion in selecting panelists. Indeed, the olfactive performance and the sensory background in qualifying flavors were the most important feature in this study. Additionally, Brand and Millot [16] showed that women have a more efficient sense of smell, particularly better sensitivity, identification skills, discrimination, memorization, etc., which may enhance the selection.

Each assessor received six samples for both olfactory and taste sessions, where three samples were from the first refining time and three others from the second one.

#### 4.3.2. Sample Preparation

**Figure 6:**
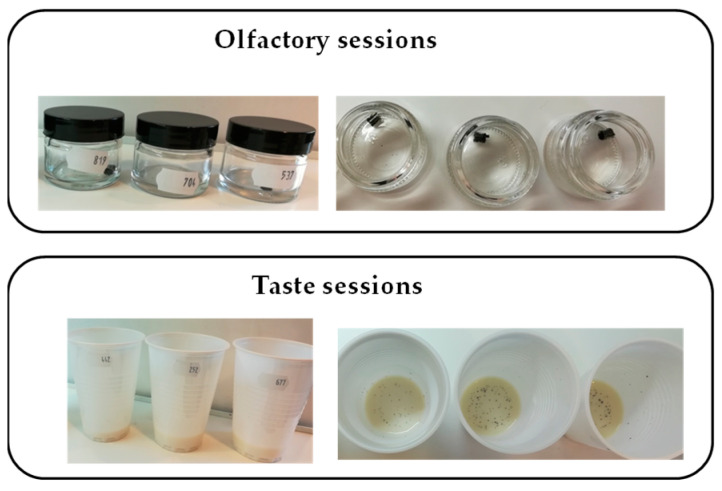
Vanilla bean sampling for olfactory and taste sessions.

Approximately 0.05 g of vanilla beans was placed in transparent vials (Figure 6) of 15 mL 72 h prior to the olfactory test.

Regarding the taste evaluation, vanilla beans were subjected to infusion within milk at a temperature of 95 °C, achieving a concentration of 0.15%. Subsequently, the vanilla-infused milk was meticulously preserved within glass vessels (Figure 6) at a constant temperature of 55 °C, pending the taste evaluation. To enhance sample uniformity, it underwent a filtration step via a fine-mesh strainer prior to its presentation to panelists.

The samples were subjected to individual assessment by panelists within a single session, comprising three samples for olfactory characterization and three samples in milk for gustatory evaluation. All samples were randomly presented to panelists.

### 4.4. Statistical Data Analysis

Statistical analyses were performed using XLSTAT^®^ software (sensory version 2021, Addinsoft, New York, NY, USA). For all the tests, measurements were performed in duplicate, and the α risk value was set at 0.05 and was compared to the *p* value obtained for each statistical analysis. Factorial correspondence analysis (FCA) was used to analyze the samples according to the means of significantly different attributes (α = 0.05). In addition, agglomerative hierarchical clustering (AHC) was performed separately on both the chemical and sensory datasets to assess the segmentation of vanilla samples, by selecting the dissimilarity coefficient and the unweighted pair-group average linkage as the agglomeration method.

## 5. Conclusions

Vanilla has become a significant flavoring agent in the food, cosmetic, and fragrance industry thanks to its high added value in formulation. Today, controlling the quality of vanilla interests the scientific community. This study enhanced the quality control of vanilla by bringing a new insight of the sensory and chemical characterization of aromatic compounds. For this purpose, the vanilla beans were treated after harvest according to two different transformation processes, scarifying and scalding. Additionally, two different times post harvesting were considered, 2 and 9 months. These parameters highly impacted the aroma and flavor profiles of *Vanilla planifolia* and *Vanilla pompona*. Further future studies should focus on assessing other types of aromas or odors, as well as different concentrations of the compounds to determine the optimal conditions of curing processes and maturity stages. As this natural product holds a great position in the global market, studies dealing with the exploration of biotechnological approaches to increase the yield of the plant should be encouraged; at the same time, the need of the hour is the development of newer and more efficient extraction methodologies to supplement the biosynthetic efforts.

## Figures and Tables

**Table 1:** Volatile organic compounds by chemical class identified in tree vanilla beans with two refining times analyzed by HS-SPME-GC-MS.

Chemical Class	Retention Times (min)	CAS	Compound	Qualitative Score (%)	Experimental RI	Theorical RI	1st Refining Time			2nd Refining Time		
							Planifolia Scalded	Planifolia Scarified	Pompona	Planifolia Scalded	Planifolia Scarified	Pompona
Alkenes	11.61	1002-33-1	1,3-Octadiene	97	979	-	-	-	-	60,270,295	65,029,884	7,245,927
Alcohols	7.05	513-85-9	2,3-butane diol	91	789	782	-	33,137,879	-	136,358,838	25,306,4021	23,827,908
	11.68	3391-86-4	1-Octen-3-ol	90	981	982	36,565,519	13,219,404	-	-	-	-
	13.24	100-51-6	Benzyl alcohol	96	1042	1041	-	-	500,967,212	107,315,093	73,052,932	218,0745,554
	15.22	60-12-8	Phenyl ethyl alcohol	97	1120	1119	-	-	16,340,937	9,382,813	14,692,848	68,511,032
	19.36	105-13-5	Para-anisyl alcohol	98	1292	1293	20,738,109	6,400,360	1,907,265,470	158,966,452	16,159,242	4,001,937,982
Aldehydes	7.39	66-25-1	Hexanal	96	806	805	35,032,094	23,017,525	-	-	-	-
	8.02	98-01-1	Furfural	91	833	836	-	-	-	58,204,606	83,405,157	13,965,141
	11.14	18829-55-5	(E)-2-heptenal	95	960	960	13,434,318	-	11,298,503	-	-	-
	11.32	100-52-7	Benzaldehyde	96	967	969	-	-	19,515,496	26,820,409	21,599,693	54,071,058
	14.84	124-19-6	Nonanal	91	1104	1106	4,710,052	3,593,206	4,634,984	10,853,957	9,782,297	9,011,181
	18.79	123-11-5	Para-anisaldehyde	97	1268	1264	36,238,748	24,790,274	259,945,096	417,431,889	134,123,512	966,387,258
	23.55	120-14-9	veratraldehyde	98	1485	1486	-	-	4,875,837	8,086,468	-	19,579,042
Aliphatic acids	4.59	64-19-7	Acetic acid	91	623	630	-	-	-	353,318,529	503,543,733	286,679,209
Ketone	5.69	513-86-0	Acetoin	90	714	714	-	-	-	17,547,670	166,297,003	
	18.44	99-49-0	Carvone	97	1253	1252	-	-	-		10,516,637	18,420,228
Esters	12.8	104-93-8	Para-methyl anisole	98	1025	1026	11,360,911	6,723,591	18,794,801	-	10,986,433	82,849,459
	14.24	104-57-4	Benzyl formate	98	1081	1079	-	-	-	7,338,858	5,629,327	6,210,826
	16.34	140-11-4	Benzyl acetate	96	1165	1167	-	-	3,210,346	8,150,143	-	27,753,414
	17.23	119-36-8	Methyl salicylate	97	1201	1201	6,013,489	4,829,471	24,056,752	18,274,367	17,768,206	49,218,129
	20.44	122-91-8	Para-anisyl formate	96	1340	-	-	-	12,713,380	-	-	10,463,838
	21.37	121-98-2	Methyl para-anisate	97	1382	-	-	-	4,713,165	5,709,997	5,230,866	16,129,396
Furane	5.48	3208-16-0	2-ethyl furan	95	702	700	-	-	-	30,458,152	9,167,432	-
	9.36	4466-24-4	2-n-Butyl furan	91	890	885	-	-	-	8,495,781	6,761,053	-
	11.92	3777-69-3	2-pentyl furan	95	991	992	11,281,928	17,262,631	-	257,807,804	72,649,944	-
Lactone	13.67	695-06-7	γ-hexalactone	90	1059	1058	-	-	6,872,802	-	-	25,753,310
Monoterpenes	13.03	5989-27-5	Limonene	98	1034	1033	29957192	-	-	-	5,261,748	6,377,838
Phenols	14.07	106-44-5	p-cresol	95	1074	1080	-	-	-	56,990,171	47,756,312	-
	14.5	90-05-1	o-guaiacol	95	1091	1093	-	20,819,785	-	174,563,068	264,653,834	38,901,742
	17.07	93-51-6	4-methyl guaiacol	97	1195	1093	-	9,975,733	20,071,774	45,669,218	42,934,133	41,218,136
	22.14	121-33-5	Vanillin	97	1413	1405	280,008,221	746,086,769	100,522,953	4,663,674,388	5,795,730,873	425,044,157

**Table 2:** A summary of the number of generated terms by 50 panelists after smelling vanilla samples.

	All Vanilla	Scalded *Vanilla planifolia*	Scarified *Vanilla planifolia*	Scarified *Vanilla pompona*
**First**				
Number of all generated terms	634	182	236	216
Number of different terms	171	42	55	74
**Second**				
Number of all generated terms	491	150	176	165
Number of different terms	187	53	62	72

**Table 3:** A summary of the number of terms generated by 50 panelists after tasting the vanilla samples.

	All Vanilla	Scalded *Vanilla planifolia*	Scarified *Vanilla planifolia*	Scarified *Vanilla pompona*
**1st**				
Number of all generated terms	772	236	281	255
Number of different terms	209	65	78	66
**2nd**				
Number of all generated terms	595	196	201	198
Number of different terms	239	82	79	78

**Table 4:** A summary of the common components between all vanillas regardless the refining time.

CAS	Compound	Odors	Flavors
105-13-5	Para-anisyl alcohol	sweet, powdery, hawthorn, lilac, rose, floral, hyacinth	cherry, vanilla, creamy nuances, cocoa, licorice, anise
124-19-6	Nonanal	waxy, aldehydic, rose, fresh, orris, orange peel, fatty, peely	creamy, powdery, vanilla, and spicy with a typical marshmallow flavor
123-11-5	Para-anisaldehyde	sweet, powdery, mimosa, floral, hawthorn, balsam	creamy, powdery, vanilla, and spicy with a typical marshmallow flavor
119-36-8	Methyl salicylate	wintergreen mint	sweet, salicylate, and root beer, with aromatic and balsamic nuances
121-33-5	Vanillin	sweet, vanilla, creamy, chocolate	vanilla, vanillin, sweet, creamy, spicy, phenolic, and milky

## Data Availability

No new data were created or analyzed in this study. Data sharing does not apply to this article. Data are contained within the article.
